# Recruitment strategies for a multisite telehealth trial for rural cancer survivors with ostomies

**DOI:** 10.1007/s00520-026-10767-y

**Published:** 2026-05-22

**Authors:** Robert S. Krouse, Stephanie B. Wheeler, Scott Appel, Virginia Sun, Matthew E. Nielsen, Rebecca L. Hoffman, Tullika Garg, Sarah M. Popek, Robert P. Sticca, Allison A. Aka, Cedrek L. McFadden, Walid M. Hesham, Marcia Grant, Michael Holcomb

**Affiliations:** 1 Department of Surgery, University of Pennsylvania Perelman School of Medicine, Leonard Davis Institute of Health Policy, Corporal Michael J. Crescenz Veterans Affairs Medical Center, 3400 Spruce St, Philadelphia, PA 19104 USA; 2 Department of Health Policy and Management, School of Medicine, University of North Carolina at Chapel Hill, Chapel Hill, NC USA; 3 Biostatistics Analysis Center, University of Pennsylvania, Philadelphia, PA USA; 4 Division of Nursing Research and Education, Department of Population Sciences, City of Hope, Duarte, CA USA; 5 Department of Urology, University of North Carolina, Chapel Hill, NC USA; 6 Previously Affiliated: Arizona Telemedicine Program, University of Arizona, Tucson, AZ USA; 7 Department of Urology, Geisinger Health System, PA Danville, USA; 8 Department of Surgery, University of New Mexico, Albuquerque, NM USA; 9 Division of Colorectal Surgery, Geisinger Medical Center, Danville, PA USA; 10 Department of Surgery, University of North Dakota, ND Grand Forks, USA; 11 Department of Surgery, Loma Linda University Health, Loma Linda, CA USA; 12 Department of Surgery, University of South Carolina School of Medicine Prisma Health-Upstate, Greenville, SC USA; 13 Division of General Surgery, Lancaster General Hospital, Lancaster, PA USA

**Keywords:** Ostomy, Rural, Telemedicine, Cancer survivors

## Abstract

**Purpose:**

Ostomies, the surgical exteriorization of bowel for urine or stool, are part of the treatment of some cancers. We completed a randomized clinical trial testing a curriculum to help cancer survivors with ostomies in rural areas delivered by telehealth. The aim of this report is to describe modifications necessary to complete accrual in this population of cancer survivors.

**Methods:**

Rural cancer survivors with ostomies at least 6 weeks postoperatively were randomized to group educational sessions via telehealth versus usual care. The curriculum was delivered once per week over 5 weeks; primary patient reported endpoints were at 6 months. Initial sites were University of North Carolina, City of Hope National Medical Center, and Geisinger Medical Center. Rurality was initially defined utilizing the rural-urban commuting area (RUCA) codes as 4+ (excluding RUCA 1, 2, 3).

**Results:**

This study was open from August 16, 2019, to February 9, 2024. Accrual was more challenging after the COVID pandemic began in March 2020. Multiple modifications were needed to address trial recruitment challenges. Our amendments included adding six sites across the USA, expanding inclusion criteria to include RUCA 3 and then RUCA 2, and adding distance from ostomy nursing care, first to those living greater than 45 miles, and subsequently more than 25 miles.

**Conclusions:**

Accrual to cancer survivorship trials may be difficult, and unpredictable circumstances such as the worldwide pandemic may require research teams to be flexible and creative with recruitment strategies. Modifications may be necessary to ensure accrual yet maintain study integrity.

**Trial registration:**

This study was registered on Clinicaltrials.gov (NCT # 03913715) on 5/29/2019.

## Introduction

An estimated 18–35% of patients diagnosed with colorectal, gynecologic, or urologic cancers will experience a temporary or permanent ostomy as part of their surgical treatments [1, 2]. While ostomy training often occurs in the perioperative setting, this can never replicate the realities of life outside the hospital. The nuances of managing an ostomy, for both patients and caregivers, typically occur by trial and error in the context of one’s day-to-day life. The myriad issues that a cancer survivor must live with subsequent to ostomy placement remain unclear. Ostomy care may be especially challenging in rural and remote areas where geographic access to specialists and ostomy support services may be limited.

Cancer patients in rural areas face multiple location and travel-associated barriers to access distant healthcare services [3]. Telehealth can overcome distance barriers by utilizing broadband internet communications and digital technologies to enable healthcare providers to deliver a wide range of healthcare services and education to patients and their informal caregivers. Telehealth service success depends on many factors such as access to reliable and affordable broadband internet service, compatible personal devices such as computers, smartphones, or tablets, and digital literacy. Organizations offering telehealth services should offer commensurate technical resources to assure that their patients have equitable opportunities to access and participate in those services [4].

Telehealth and other remote service offerings can enhance adult learning around survivorship support and ostomy care in rural areas. Adult learning is defined as how adults obtain new knowledge and skills and focuses on the belief that adults learn differently from children based on life experiences, self-directedness, and practical needs [5]. Key principles include adults setting learning goals, locating resources, relating to real-life experience, using discussions and examples, focusing applying knowledge to practical situations, and engaging intrinsic motivation factors like personal satisfaction or career development [6–8]. Therefore, it is important to consider these principles when designing a curriculum for adult learners. Adult cancer survivors face unique stressors and challenges, as well as opportunities, when learning new skills related to optimizing their health and healthcare.

Our team developed Ostomy Self-Management Training (OSMT) as a curriculum for adult postoperative cancer survivors and their support persons/caregivers [9–16]. The intervention is designed to be delivered by ostomy nurses and peer ostomates (experienced, trained cancer survivors with ostomies) in a group setting via telehealth. We have had success in delivering the intervention to patients at urban academic institutions and were interested in expanding to rural populations [16]. The purpose of this report is to outline our recruitment methods for rural cancer survivors, especially during the COVID-19 pandemic which impacted medical care and research [17].

## Methods

**Fig. 1:**
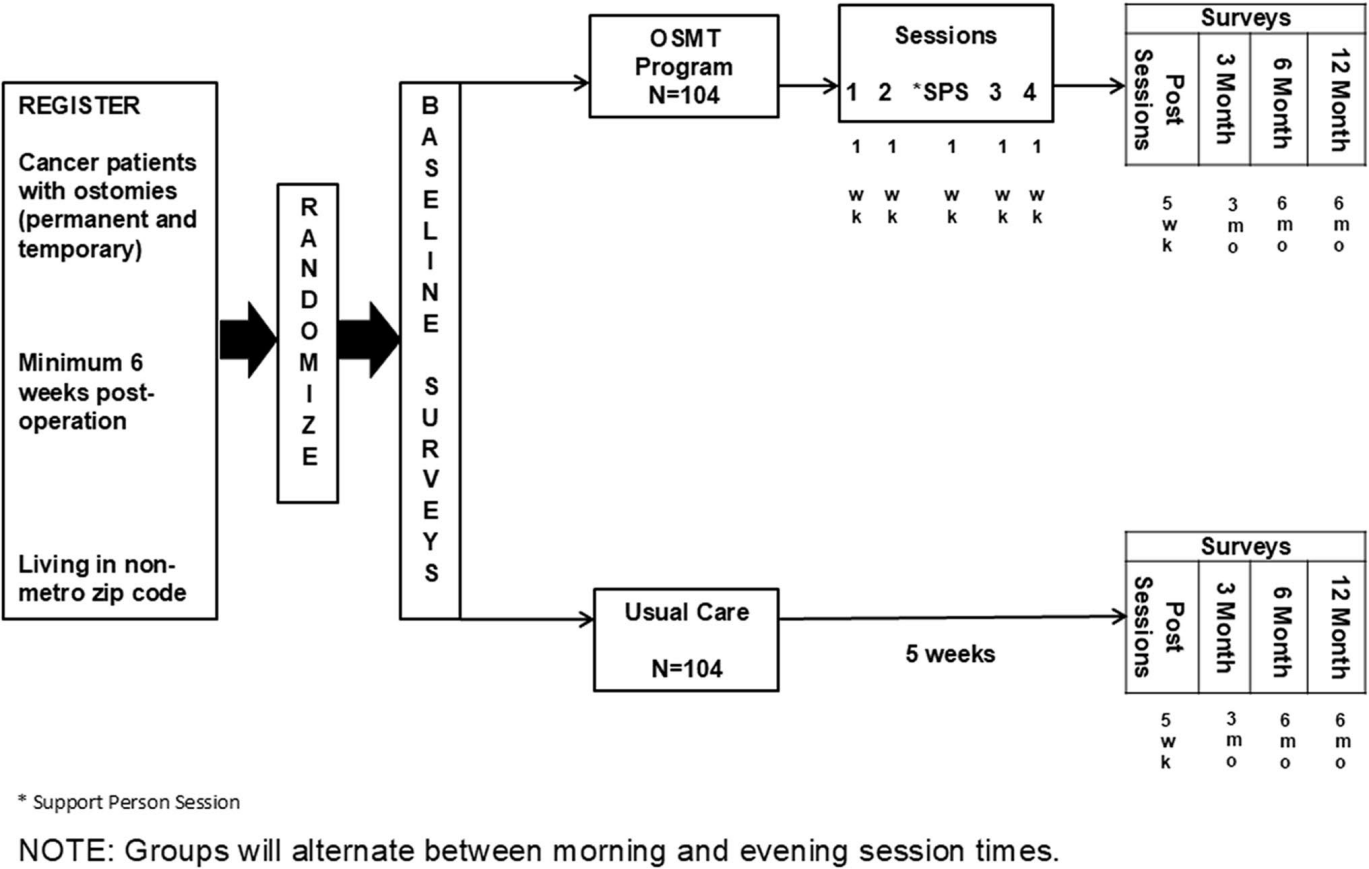
Study design. Note: Groups alternated between morning and evening session times

Rural cancer survivors at least 6 weeks from their ostomy surgery were randomized to group educational sessions delivered via telehealth (Fig. 1) or to usual care. The curriculum was delivered once per week over 5 weeks, with the third (middle) session only for informal caregivers and the fifth session for participants and informal caregivers. Initial sites were the University of North Carolina, City of Hope National Medical Center (California), and Geisinger Medical Center (Pennsylvania). The original recruitment goal from these three sites was four patients per month. With the onset of COVID-19 and the slowing of accrual, our goals shifted to having as many participants as possible per session hoping to continue to accrue to trial completion.

Rurality was defined utilizing the U.S. Department of Agriculture’s (USDA) Rural-Urban Commuting Area (RUCA) codes which classify US census tracts using measures of population density, urbanization, and daily commuting to define levels of rurality and urbanicity [18]. The codes are used to classify census tracts and ZIP codes into categories such as metropolitan (RUCA codes 1–3), micropolitan, small town and rural (RUCA codes 4–10) [19]. There are many definitions of rurality, and we selected census tract-based RUCA codes because they provide a nuanced, geographically specific definition of rural and urban areas compared to larger county-based definitions. This specificity helps to avoid heterogeneity issues associated with larger units, making RUCA codes valuable for targeting federal health care programs and other policy initiatives [20, 21].

This study protocol was approved by each site’s Institutional Review Board. The research team met weekly throughout the study period to ensure fidelity to inclusion/exclusion criteria and intervention plans. The protocol was strictly adhered to and was amended as needed through a formal modification process.

## Results

**Fig. 2:**
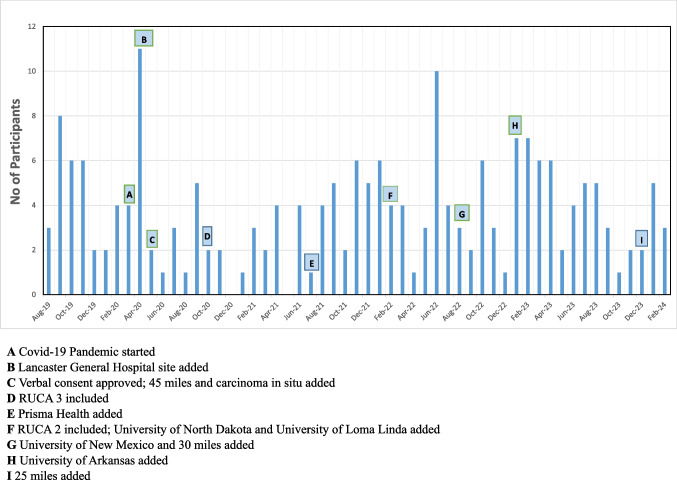
Rural study monthly recruitment 8/2019–2/2024. (A) Covid-19 Pandemic started. (B) Lancaster General Hospital site added. (C) Verbal consent approved; 45 miles and carcinoma in situ added. (D) RUCA 3 included. (E) Prisma Health added. (F) RUCA 2 included; University of North Dakota and University of Loma Linda added. (G) University of New Mexico and 30 miles added. (H) University of Arkansas added. (I) 25 miles added

A total of 569 patients met eligibility criteria and were approached to participate in the study. Of those, 365 (64.2% declined because they were not interested, technical concerns, medical or personal issues, or other reasons. Therefore, 204 (35.9%) cancer survivors with ostomies were randomized into the study from August 16, 2019, to February 9, 2024. Groups were well-matched on demographic and clinical factors (Table 1). While initial accrual was brisk, the COVID-19 pandemic, which began in March 2020, introduced recruitment challenges (Fig. 2). Related to retention, there were multiple factors for patient dropout. These included lost to follow-up (13; 25.0%), disease progression (12; 23.1%), refused surveys (11; 21.2%), no longer wanted to participate (9; 17.3%), deceased (3; 5.8%), failed post-screening (3; 5.8%), and ostomy reversal (1; 1.9%). Dropout was more common in the OSMT arm (35/104; 33.7%) than the UC arm (17/100; 17.0%). A major strength of our trial was that it was completely administered virtually. As a result, we were able to maintain fidelity to the study and the intervention while implementing modifications to our recruitment strategy to continue accrual including adding new sites, expanding the rural definition, and shifting recruitment to fully virtual via telephone.

**Table 1:** Participant characteristics (*N* = 204 subjects were randomized)

	Usual care (*N* = 100)	OSMT (*N* = 104)	*P*-value
**Gender (%)**			0.974
M	56 (56.0)	58 (55.8)	
F	44 (44.0)	46 (44.2)	
**Age**			0.938
Mean (SD)	64.0 (13.1)	63.9 (13.5)	
Median	64.0	65.0	
**Race (%)**			0.979
American Indian/Alaska Native	1 (1.0)	0 (0.0)	
Asian	1 (1.0)	1 (1.0)	
African-American	8 (8.0)	8 (7.7)	
Caucasian	72 (72.0)	66 (63.5)	
More than one race	0 (0.0)	1 (1.0)	
Native Hawaiian or Pacific Islander	1 (1.0)	1 (1.0)	
Other	7 (7.0)	5 (4.8)	
Unknown/missing	10 (10.0)	22 (21.2)	
**Ethnicity (%)**			0.652
Hispanic	13 (13.0)	9 (8.7)	
Non-Hispanic	73 (73.0)	64 (61.5)	
Unknown/missing	14 (14.0)	31 (29.8)	
**Marital status (%)**			0.566
Partnered	65 (65.0)	61 (58.7)	
Non-partnered	26 (26.0)	20 (19.2)	
Unknown	9 (9.0)	23 (22.1)	
**Type of cancer (%)**			0.908
Colorectal	46 (46.0)	47 (45.2)	
Urological	54 (54.0)	57 (54.8)	
**Stoma type (%)**			0.630
Colostomy	33 (33.0)	34 (32.7)	
Urostomy	29 (29.0)	23 (22.1)	
Ileostomy	19 (19.0)	14 (13.5)	
More than one ostomy	2 (2.0)	4 (3.8)	
Unknown	17 (17.0)	29 (27.9)	
**Education (%)**			0.353
Did not complete HS	8 (8.0)	8 (7.7)	
Completed high school	27 (27.0)	14 (13.5)	
Vocational/secretarial	4 (4.0)	2 (1.9)	
Some college	26 (26.0)	18 (17.3)	
College degree	13 (13.0)	19 (18.3)	
Some grad school	2 (2.0)	3 (2.9)	
Graduate degree	11 (11.0)	13 (12.5)	
Unknown	9 (9.0)	27 (26.0)	
**Employment (%)**			0.863
Employed			
Yes	14 (14.0)	15 (14.4)	
No	5 (5.0)	2 (1.9)	
Retired	40 (40.0)	37 (35.6)	
Home maker	6 (6.0)	3 (2.9)	
Volunteer	2 (2.0)	2 (1.9)	
Disabled	12 (12.0)	12 (11.5)	
Unknown/Missing	21 (21.0)	33 (31.7)	
**Income (%)**			0.729
Less than 30,000	36 (36.0)	27 (26.0)	
30,000—75,000	27 (27.0)	25 (24.0)	
Greater than 75,000	25 (25.0)	25 (24.0)	
Unknown	12 (12.0)	27 (26.0)	
**Smoking history (%)**			0.032
Currently smoker	11 (11.0)	8 (7.7)	
Never smoked	28 (28.0)	39 (37.5)	
Quit smoking	50 (50.0)	29 (27.9)	
Unknown	11 (11.0)	28 (26.9)	
**Time since ostomy surgery (%)**			0.493
< 6 months	62 (62.0)	58 (55.7)	
6 months- 2 years	25 (25.0)	27 (26.0)	
> 2 Years	11 (11.0)	17 (16.3)	
Unknown	2 (2.0)	2 (1.9)	
**BMI**^**a**^** (%)**			0.697
Underweight	5 (5.0)	7 (6.7)	
Normal	33 (33.0)	30 (28.8)	
Overweight	48 (48.0)	37 (35.6)	
Obesity	6 (6.0)	3 (2.9)	
Unknown	8 (8.0)	27 (26.0)	

^a^Underweight: BMI < 18.5, normal 18.5–24.9, overweight 25–39.9; obesity: BMI >  = 40

Sites were added throughout the study with the goal of ensuring a range of geographic sites in the USA. We added the following sites over the course of the trial: Loma Linda University Health (California), University of South Carolina Greenville (Prisma Health), Sanford Medical Center (North Dakota), University of New Mexico, Lancaster General Hospital (Pennsylvania), and University of Arkansas. The University of Pennsylvania was the central IRB of record; one challenge we faced were long timelines to initiate accrual at new sites due to IRB and contracting processes.

The definition of rurality was expanded from RUCA 4+ to include other patient populations in residential areas that were not defined as metropolitan core (i.e., RUCA 1), notably including patients in RUCA 2 and 3 census tracts. While this limited our ability to continue to label the target patient population “rural,” it did remain a non-metropolitan cohort. We also added patients who lived greater than 45 miles from an ostomy nurse or a medical center that had an in-house ostomy nurse. We later decreased the distance to 25 miles to expand access further to those survivors in need of ostomy care. Most participants were considered rural via RUCA criteria (60%; 123/204); 38% (80/204) were RUCA 2 or 3 (Table 2). Of those who lived in Metropolitan RUCA codes, 96% (78/81) were at more than 25 miles, and most lived at substantially greater distances from ostomy nurses (mean 81.6miles, median 69.9 miles).

**Table 2:** Rurality of study participants

Miles to the nearest ostomy nurse	Overall *N* (%)	Metropolitan* N (%)	Micropolitan** N (%)	Small town/rural*** N (%)
0 to 25	27 (13.2%)	3 (4.2%)	21(38.2%)	3 (13.6%)
> 25 to 50	48 (23.5%)	31(43.7%)	6 (10.9%)	11 (50.0%)
> 50 to 75	37 (18.1%)	24 (33.8%)	12 (21.8%)	1 (4.5%)
> 75 to 100	39 (19.1%)	11 (15.5%)	21 (38.2%)	7 (31.8%)
> 100	50 (24.5%)	12 (16.9%)	25 (45.4%)	13 (59.2%)
Total	204	81	88	35

^*^RUCA 1, 2, and 3; **RUCA 4, 5, and 6; ***RUCA 7, 8, 9, and 10

3 subjects in micropolitan group were missing distance to the ostomy nurse

The most significant challenge to recruitment that we encountered was related to COVID-19. Institutional changes to patient visits and potentially surgical care were altered during this time. Therefore, we modified to virtual-only recruitment methods early in the COVID-19 pandemic as study coordinators were not allowed in the clinics to conduct in-person recruitment, which continued for much of the accrual period for this study. This was allowed for the remainder of the study, although coordinators did also see patients in clinics late in the accrual process as COVID restrictions eased; the timing was variable at each institution. Patient recruitment letters were added as an enrollment tool and phone scripts were included to ensure conformity of messaging. The team considered adding Spanish-only speakers as there were multiple rural cancer survivors in the Loma Linda catchment area that would have been eligible. We opted not to allow translators as sessions 1–3 were designed solely for participants.

Another challenge was that many patients experienced disease progression or were under active treatment. This made retention on the study more difficult. Some participants also faced health declines requiring hospitalization or transition to hospice care leading to multiple patients dropping out.

## Discussion

Rural cancer survivors with ostomies often have unmet ostomy care and other treatment-related needs. This may often be related to the distance necessary to travel to specialized ostomy care resources. We designed a study to test a patient-centered group training intervention versus usual care in this patient population. The goal of utilizing telehealth was to eliminate travel and associated burdens. Accrual to this study, especially during the COVID-19 pandemic, was challenging and mandated the research team to continually reassess strategies to engage this cancer survivor population. This included refining definitions of rural populations and implementing virtual patient accrual processes.

Our approach in this study included proactively working with intervention participants in advance of scheduled OSMT sessions to ensure that they could successfully connect to, and participate in discussions via, the secure cloud video conferencing platform. While most of the participants in this study had their own technology and internet access, some participants required loaned devices with mobile data plans to facilitate their participation. More than 20% of all households in rural areas lack reliable internet access [22]. Non-metropolitan area households are more likely to experience limitations in devices and internet connectivity [23].

The COVID-19 pandemic spurred tremendous growth in telehealth adoption and utilization while also adversely affecting the accrual of study participants due to non-telehealth-related factors [24]. There are many reasons why the COVID-19 pandemic impacted accrual for this study, including limited diagnostic procedures, research staff no longer allowed in clinics, changes in patient flow, and other factors. First, hospital regulations made diagnostic testing with endoscopy, cystoscopy, radiologic testing, and many other forms of testing unavailable, delayed, or limited for long periods of time [25–30]. Surgical procedures were often delayed. Patterns of care, such as certain hospitals in a system focusing care more than others, may have been instituted. Research teams were mandated to work from home. Therefore, even though our telehealth study should have been set up for success in the COVID-19 pandemic, many factors made accrual more challenging.

The rural focus of this study developed from a previous urban- and academic-medical center focused study on cancer survivors with ostomies [16]. It is likely that there is a need of services beyond those categorically labelled “rural” through RUCA 4+ classification. Importantly, there is not a single definition used across all federal agencies. Each agency creates their own process to define rurality for their purposes. For our study, we used the U.S. Department of Agriculture’s (USDA) RUCA framework, which uses population density and commuting patterns to assign these designations to census tracts [18, 31]. These measures offer more detailed insight into how far rural communities are from health care services in more populated areas. Throughout our study and through engagement with clinical partners, we learned that limiting enrollment to RUCA 4+ would unnecessarily restrict access to ostomy care that other non-metropolitan core dwelling survivors needed. Thus, we broadened our inclusion criteria to address the needs of others with poor access.

This study ultimately focused on non-metropolitan core populations by current RUCA definitions, but the spirit of cancer survivors who have geographic related difficulties in ostomy care access was upheld. Importantly, OSMT participants were able to meet and discuss issues with others who had similar obstacles to care. Our study team adapted to changing conditions to ensure continued accrual as difficulties were encountered. A limitation may be the exclusion of Spanish-only speakers, which would have added to our diversity and rapidity to complete the study. In the future, the OSMT program may be revised to ensure that the intervention is relevant to Spanish-speakers, and pilot-tested to ensure feasibility. The need to train ostomy nurses and peer ostomates that are Spanish speakers will ensure that the program and its content is delivered as intended and not “lost in translation.” An alternative, reasonable option is to include small Spanish-only groups, or inclusion of translators.

Cancer survivors, especially those undergoing active therapy or more advanced disease, are a vulnerable group. We did actively reach out to participants who experienced cancer-related reasons for dropping out from the study, but physical limitations often precluded continuing in the study. Our study was designed to include all patients with cancer and an ostomy. We believe it is important to be as inclusive as possible.

The OSMT study for cancer survivors with ostomies was successful in completing accrual in a time of great stress to medical systems and research teams. It displays the importance of working with multidisciplinary teams to continually think through problems, and adapt where possible, to ensure completion of study with appropriate data collection. While a pandemic is unlikely to be repeated, clinical trial recruitment of patients in rural areas will continue to pose challenges to recruitment, and clear methods to overcome such barriers are imperative. This included constant reassessment of recruitment challenges and means of addressing them. While this may entail changes in inclusion criteria, the essence of the study must be maintained.

There are also several insights that can assist future researchers who wish to initiate a similar trial. First, while each site kept its own recruitment log, this could be standardized to ensure all recruitment and retention efforts are the same. Second, augmenting the use of peers in the recruitment and retention process may have helped; while we encouraged peers reach out to potential participants, this was not part of the standardized protocol, and it could be in the future. Another recommendation centers on data collection. While the patient-reported outcomes (PRO) included in the study were important mandatory outcomes, some participants felt they were burdensome. During the recruitment process, some potential participants did not want to fill out our compendium of surveys over time. While we did encourage completing the primary outcome survey first, it is possible to standardize PRO priorities both in recruitment and retention. Finally, introducing the study prior to their ostomy procedure or soon after may have made potential patients more willing to participate in the study. Being familiar with the study staff and the purpose of the study is likely to make people more amenable to hearing about it at a later date.

## Conclusions

Accrual to clinical trials for surgical patients is typically challenging but was particularly difficult during the COVID-19 pandemic. Factors outside of control may impact accrual. It is important to adapt to changing conditions to ensure study completion. For our study, while our population may not be entirely “rural” by all definitions, the spirit of our amendments was to promote access to ostomy care for all who need it in non-metropolitan areas, while ensuring that accrual goals could be met.

## Data Availability

No datasets were generated or analysed during the current study.

## Declarations

### Disclaimer

This content is the author’s responsibility and does not represent the NIH’s official views.
